# Psoas Muscle Area Predicts Mortality after Left Ventricular Assist Device Implantation

**DOI:** 10.3390/life11090922

**Published:** 2021-09-05

**Authors:** Franziska Wittmann, Thomas Schlöglhofer, Julia Riebandt, Anne-Kristin Schaefer, Dominik Wiedemann, Edda Tschernko, Dietrich Beitzke, Christian Loewe, Günther Laufer, Daniel Zimpfer

**Affiliations:** 1 Division of Cardiac Surgery, Medical University of Vienna, 1090 Vienna, Austria; franziska.wittmann@meduniwien.ac.at (F.W.); thomas.schloeglhofer@meduniwien.ac.at (T.S.); julia.riebandt@meduniwien.ac.at (J.R.); anne-kristin.schaefer@meduniwien.ac.at (A.-K.S.); dominik.wiedemann@meduniwien.ac.at (D.W.); guenther.laufer@meduniwien.ac.at (G.L.); 2 Center for Medical Physics and Biomedical Engineering, Medical University of Vienna, 1090 Vienna, Austria; 3 Ludwig Boltzmann Institute for Cardiovascular Research, 1090 Vienna, Austria; 4 Division of Cardiac Thoracic Vascular Anesthesia and Intensive Care Medicine, Medical University of Vienna, 1090 Vienna, Austria; edda.tschernko@meduniwien.ac.at; 5 Division of Cardiovascular and Interventional Radiology, Medical University of Vienna, 1090 Vienna, Austria; dietrich.beitzke@meduniwien.ac.at (D.B.); christian.loewe@meduniwien.ac.at (C.L.)

**Keywords:** mechanical circulatory support, risk stratification, sarcopenia, frailty

## Abstract

Several risk scores and classifications are available to predict peri- and post-operative mortality of patients with end stage heart failure receiving Left Ventricular Assist Device (LVAD) therapy. Sarcopenia has been suggested as a sensitive predictor for post-operative mortality. We evaluated whether the psoas muscle area can predict mortality in patients undergoing LVAD implantation. The indexed psoas mean area (PMAi) was obtained by measuring the psoas muscle area at the superior endplate of the third lumbar vertebra correlated to body surface area of 106 adult patients undergoing LVAD implantation (Medtronic HVAD *n* = 41, Abbott HeartMate II *n* = 4, Abbott HeartMate 3 *n* = 61; mean age 65, IQR 12, 90.6% male; INTERMACS Level 1 24.5%; ischemic CMP 64.2%). Patients were divided in two groups: high/moderate and low muscle mass. The primary endpoint was 30-day mortality, assessed using a multivariate Cox proportional hazards model. Baseline characteristics did not differ between patients with high or moderate and low PMAi. Estimated survival calculated a significant higher 30-day mortality in patients with low PMAi (*p* = 0.04). Multivariable Cox proportional hazards regression analysis indicated low PMAi, history of previous cardiac surgery and levels of bilirubin as independent predictors of mortality in the first 30 days. In conclusion, indexed psoas muscle area predicts mortality after LVAD implantation and can be used as an additional tool for risk stratification.

## 1. Introduction

Cardiovascular diseases (CVDs) are the number one cause of morbidity and mortality in today’s society [1]. Terminal heart failure (HF) is often the ultimate fate of patients suffering from CVDs. Available treatment options include conservative medical therapy, heart transplantation and implantation of a left ventricular assist device (LVAD) [2]. Survival after LVAD implantation has improved significantly over recent decades [3]. This progress has been achieved through technical improvements in the implantable devices as well as improved peri- and post-operative patient management. Furthermore, patient selection has proven to be crucial for outcomes and thus careful risk stratification is needed to evaluate the benefit for each individual patient. The INTERMACS classification was introduced to evaluate post-operative outcomes for patients with terminal HF undergoing LVAD implantation. The classification categorizes patients from level 1, representing patients in cardiogenic shock, thus the sickest cohort, to level 7, which represents the ambulatory HF patient without recurrent fluid imbalances [4]. The classification is most commonly used to estimate mortality within one year after LVAD implantation. However, to further refine patient selection, other potential markers are being evaluated. Most recently, sarcopenia has been suggested as sensitive predictor for post-operative mortality. The rationale of this hypothesis is that the amount of muscle mass reflects the patient’s resources and thus influences post-operative outcome [5,6]. Preserved muscle strength has been shown to correlate with survival in patients with HF [7]. Recent studies investigating whether or not sarcopenia can be used as a risk factor in patients destined for transcatheter aortic valve implantation showed that reduced muscle mass increased post-interventional mortality [8,9]. Therefore, the aim of this study was to evaluate if muscle mass can serve as an independent predictor for mortality after LVAD implantation.

## 2. Materials and Methods

The study was approved by the Ethics Committee of the Medical University of Vienna (EK no.: 1205/2020; 31 March 2020).

### 2.1. Patient Population

**Figure 1:**
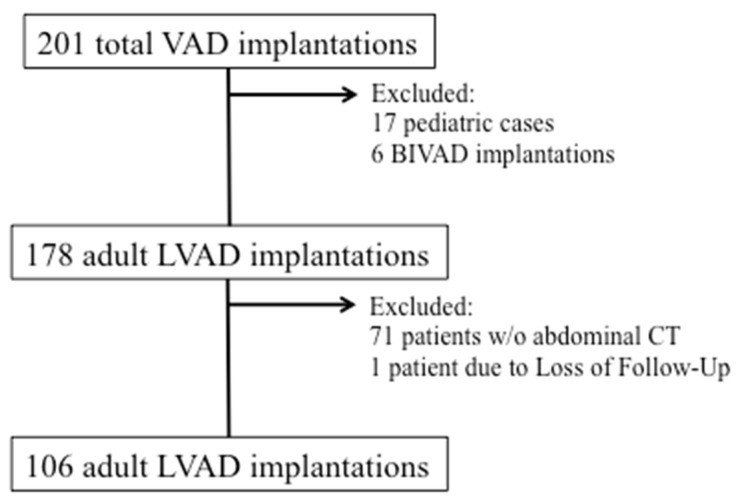
Patient Flow Chart. In the time period from January 2014 to April 2019 a total of 201 durable ventricular assist devices were implanted at our center. Of these, 106 adult patients met all inclusion criteria and had a pre-operative abdominal CT scan available for measurement of the psoas muscle.

We retrospectively analyzed data of 201 patients receiving durable ventricular support from January 2014 to April 2019 at the Division of Cardiac Surgery, Medical University of Vienna. Patients receiving permanent biventricular support, patients below 18 years of age and patients with loss of follow up were excluded. The remaining patients were screened for availability of a preoperative CT scan of the abdomen including the psoas muscle in the time span 2 weeks prior to surgery. These 106 adult patients entered the analysis (Figure 1).

### 2.2. Measurement of Indexed Psoas Mean Area (PMAi)

**Figure 2:**
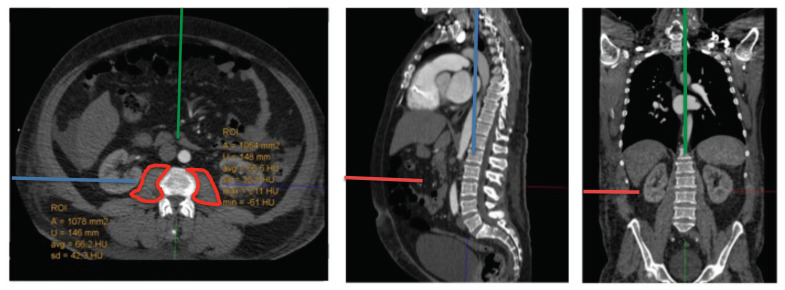
Measurement of Psoas Mean Area. Psoas Mean Area was measured (circled in red) in an axially corrected fashion at the level of the superior endplate of the third lumbar vertebra.

Psoas mean area was measured at the level of the superior endplate of the third lumbar vertebra by outlining the circumference of the right and left psoas muscle in an axially corrected fashion using IMPAX Software (Version EE (R 20 XIX), Agfa HealthCare, Mortsel, Belgium). (Figure 2) Indexed psoas mean area (PMAi) was calculated by correlating the psoas mean area with patient’s body surface area (BSA) using the Dubois formula (BSA = 0.007184 ∗ Height^0.725^ ∗ Weight^0.425^) [10]. Patients then were divided in two groups: high or moderate muscle mass and low muscle mass in accordance with patient sex as previously described (high or moderate: women, >635 mm^2^/m^2^; men, >856 mm^2^/m^2^; low: women, ≤635 mm^2^/m^2^; men, ≤856 mm^2^/m^2^) [8].

### 2.3. Study Outcomes

The primary end point was 30-day post LVAD mortality. Secondary endpoints were length of ICU stay, length of catecholamine support and number of ventilation days.

### 2.4. Statistical Analyses

Descriptive statistics are presented as mean ± standard deviation (SD) for continuous variables and number (percentage) for categorical variables. Where continuous variables were non-normally distributed, data are presented as median and interquartile range (IQR). Normal distribution was assessed by the Shapiro-Wilk test. Data were analyzed for a Gaussian distribution and consequently subjected either to parametric tests (*t*-test or one way ANOVA), non-parametric tests (Mann-Whitney U Test or Kruskal-Wallis Test) or Pearson’s χ^2^ or Fisher’s exact test for categorical variables using SPSS software (Version 26, IBM, Armonk, NY, USA). Survival curves were estimated and depicted using the Kaplan–Meier method and tested by the log-rank test. Patient follow-up was censored when patients underwent heart-transplantation, device explantation, or expired. A stepwise multivariate Cox proportional hazards model for 30 day mortality was constructed with criteria for entry of *p* < 0.1 and staying threshold of *p* < 0.05. Based on the results of the univariable analysis the following variables were entered into the baseline multivariable model: group PMAi, INTERMACS profile, prevalence of pulmonary hypertension, redo cardiac surgery, bilirubin levels and cholinesterase levels.

## 3. Results

### 3.1. Patient Characteristics

Mean patient age was 65 (IQR 12) years, the majority of patients (90.6%) were male and 41.5% were in INTERMACS Levels 1 and 2. The majority of patients had ischemic cardiomyopathy (64.2%, *n* = 68), 36 (34.0%) patients had dilative cardiomyopathy and two (1.9%) of the patients had an unidentified etiology. Forty-one patients (38.7%) received an HVAD (Medtronic Inc., Minneapolis, MN, USA), four patients (3.8%) a HeartMate II (Abbott Inc., Chicago, IL, USA) and 61 (57.5%) patients a HeartMate 3 (Abbott Inc., Chicago, IL, USA) device. Detailed patient characteristics are given in Table 1.

The patient cohort consisted of 81.1% (*n* = 86) patients with high or moderate and 18.9% (*n* = 20) patients with low muscle mass, with a median PMAi of 992.34 (IQR 269.6) mm^2^/m^2^ and 578.3 (IQR 138.8) mm^2^/m^2^ (*p* < 0.001) respectively. Besides PMAi, patients with high or moderate and low muscle mass were comparable with regard to preoperative patient characteristics including INTERMACS level (Table 1).

### 3.2. Impact of PMAi on Mortality

**Figure 3:**
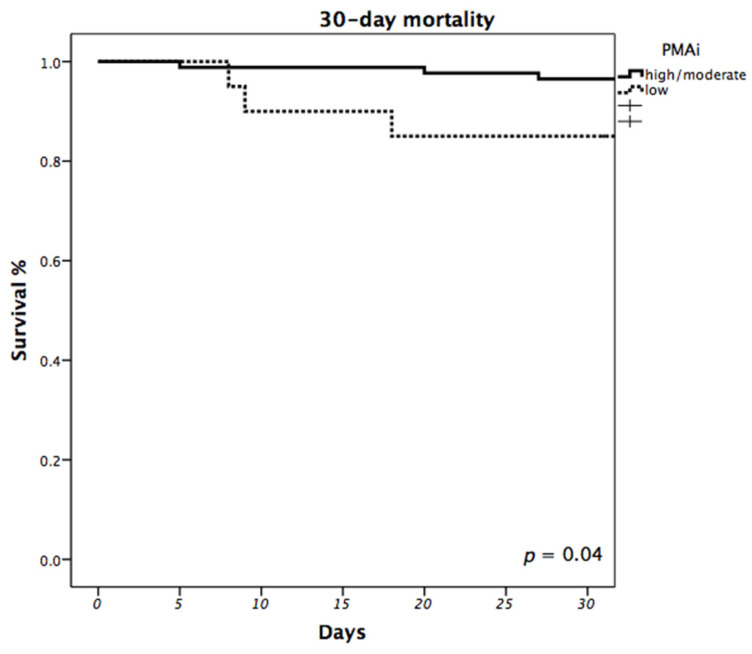
Impact of PMAi on 30-day Mortality. Estimated 30-day survival was calculated using the Kaplan-Meier estimator and compared between patients with low and high/moderate PMAi. (*p* = 0.04).

Overall 30-day mortality was 5.7%. When comparing patients with different PMAi, observed 30-day mortality was significantly higher in patients with low muscle mass (15%) compared to patients with high/moderate muscle mass (3.5%; *p* = 0.045). Estimated 30-day survival calculated using the Kaplan-Meier estimator also showed higher mortality in patients with low PMAi (*p* = 0.04; Figure 3). Multivariable Cox proportional hazards regression analysis indicated that low PMAi (high/moderate vs. low, HR: 27.3, 95% CI: 2.736–272.797; *p* = 0.005), history of previous cardiac surgery (HR: 29.8, 95% CI: 1.894–467.644; *p* = 0.016) and levels of bilirubin (HR: 1.426, 95% CI: 1.093–1.860; *p* = 0.009) were independent predictors of mortality in the first 30 days after LVAD implantation (Table 2).

### 3.3. Impact of PMAi on Post-Operative Intensive Care Effort

Furthermore, the impact of pre-operative muscle mass on post-operative effort on the ICU was evaluated. Therefore, length of catecholamine support, ventilation times and ICU stay were compared. Mean length of ICU stay in the patient cohort was 11 days (20); mean length of catecholamine support was 7 (12) days and mean length of ventilation was 1 (IQR 3) day respectively. Comparing high/moderate and low PMAi, no statistical differences were found, neither in length of ICU stay: 11 (IQR 21) vs. 17 (IQR 16) (*p* = 0.288), in days of catecholamine support: 7 (IQR 12) vs. 9 (IQR 11) (*p* = 0.446), nor in length of ventilation: 1 (IQR 2) vs. 5 (IQR 9) (*p* = 0.096). Incidences of post-operative complications based on the INTERMACS adverse event definitions were also evaluated. There were no statistically significant differences comparing patients with either high/moderate or low PMAi (Table A1). Causes of death were comparable between the two groups. In patients with high or moderate PMAi, one patient (1.2%) died of sepsis, one patient (1.2%) died of multi-organ-failure and one patient (1.2%) due to neurological dysfunction. In patients with low PMAi, one patient (5%) died of sepsis, one patient (5%) died of multi-organ-failure and one patient (5%) due to neurological dysfunction.

## 4. Discussion

The main finding of this study is that psoas muscle area measured in routine preoperative CT scans is an independent and easily assessable predictor for perioperative mortality in patients undergoing LVAD implantation. Other independent predicators were redo-surgery and elevated preoperative bilirubin levels. Psoas muscle area, however, did not predict post-operative ventilation times, ICU stay and duration of inotropic support. Risk factors for adverse outcomes in patients undergoing LVAD implantation have been extensively studied. Amongst others, lower INTERMACS level, advanced age, bilirubin, redo-surgery, reduced RV-function, preoperative ventilation and presence of short term mechanical circulatory support have been identified as risk factors for mortality in multiple studies [11,12]. While these risk factors define the individual patients, risk based on end-organ status, suitability for LVAD support and overall preoperative state, they do not reflect frailty. Frailty and sarcopenia are increasingly recognized risk factors for perioperative and periprocedural mortality in patients undergoing different surgical and interventional procedures. A variety of factors influence an individual patient’s frailty. Sarcopenia is one of these factors and can, for example, be quantified by measurement of muscle strength. Chung et al. reported the association of reduced hand grip strength and increased mortality after LVAD implantation [13]. Gait speed was also shown to predict post-operative outcome [14,15]. Assessment of muscle strength however is limited in intubated patients. Hence, measurement of muscle mass area in CT scans has been introduced as objective marker for sarcopenia. Multiple studies were already able to demonstrate the predictive value of psoas muscle area on post-interventional mortality in patients receiving transcatheter aortic valve implantation (TAVI) [8,9]. Yamashita et al. was able to reproduce these findings in patients undergoing cardiac surgery [16]. The applicability of muscle mass determined in CT scans as predictive marker for mortality has also already been evaluated in patients undergoing LVAD implantation [17,18]. Based on these findings, the aim of this retrospective analysis of our patient cohort was to evaluate the applicability of psoas muscle mass as additional tool for risk stratification. We measured the psoas muscle area at height of the third lumbar vertebra and categorized the patients as either having high/moderate or low muscle mass based on psoas muscle area in relation to body surface area giving PMAi. We found no relevant significant differences in baseline characteristics, such as age or comorbidities. Influence of PMAi on mortality after LVAD implantation was evaluated and patients with low compared to high or moderate PMAi had a significantly higher mortality in the first 30 days after LVAD implantation. Using multivariable Cox proportional hazards modeling, we were able to identify PMAi as an independent risk factor for short-time mortality after LVAD implantation. However, the confidence intervals are rather wide, which is due to the relatively small sample size. These results are comparable to findings by Heberton et al., who observed significantly higher in-hospital mortality of patients with low psoas muscle mass [17]. Teigen et al. evaluated pectoralis muscle mass as indicator for sarcopenia and also observed higher mortality after LVAD implantation in those patients with lower muscle mass [18]. Our study is the first to look further at influence of muscle mass on post-operative complications and ICU effort. This analysis was based on the hypothesis that patients with lower muscle mass have fewer reserves and thus have a tendency to need longer recovery times on the ICU. However, analysis of post-operative intensive care effort showed no significant differences neither in length of ICU stay, catecholamine support, nor in length of ventilation. No differences in morbidity in the perioperative setting were observed. Admittedly, our sample size seems too small to capture possibly existing differences in post-operative ICU effort and complications. The number of observed deaths in our cohort is also small, with only three deaths in each group. These results show the overall low mortality at our center and yet we were able to show that low PMAi is an independent risk factor for mortality. There were no differences in type of death observed between high/moderate and low PMA. However, comparing six deaths is too small a number to make assumptions about the effect of PMAi on type of death.

## 5. Conclusions

In conclusion, patients with low PMAi in our cohort had statistically significant higher short-term mortality. These findings show that PMAi is an independent risk factor for mortality in the first 30 days after LVAD implantation. Our study suggests that PMAi can be used as an additional tool to identify frail patients and should be taken into consideration during risk stratification preoperatively in these patients.

## 6. Limitations

The results of this study are limited as it is a retrospective analysis of single center data and consists of a rather small patient population. Moreover, not all patients who received an LVAD in the respective time span received an abdominal CT scan preoperatively, which possibly led to a skewed patient cohort and further reduced sample size. Therefore, further studies with a larger patient population, and also a larger proportion of female patients, are needed to confirm these results.

## Figures and Tables

**Table 1:** Baseline Characteristics prior to LVAD implantation. *p*-value calculated between high/moderate and low muscle mass using t-test, chi-square test or Fisher’s exact test with Monte Carlo approximation, as appropriate. Data presented as *n* (%) or mean ± standard deviation for normally distributed data or as the median with the interquartile range (IQR) for non-normally distributed data. Abbreviations: INTERMACS, Interagency Registry for Mechanically Assisted Circulation Support; BTT, Bridge to Transplant; BTC, Bridge to Candidacy; DT, Destination Therapy; BMI, Body Mass Index; HT, Hypertension; COPD, Chronic Obstructive Pulmonary disease; PVD, Peripheral Vascular Disease; PMAi, indexed Psoas Muscle Area.

Variable	Total Population (*n* = 106)	High/Moderate PMAi (*n* = 86)	Low PMAi (*n* = 20)	*p*-Value
Sex				
male, *n* (%)	96 (90.6)	79 (90.7)	18 (90)	0.92
female, *n* (%)	10 (9.4)	8 (9.3)	2 (10)	
Age at implant	65 (IQR 12)	65 (IQR 12)	63 ± 9	0.80
INTERMACS profile, *n* (%)				
1	26 (24.5)	18 (20.9)	8 (40)	0.24
2	18 (17)	14 (16.3)	4 (20)	
3	16 (15.1)	13 (15.1)	3 (15)	
4	42 (39.6)	38 (44.2)	4 (20)	
5	3 (2.8)	2 (2.3)	1 (5)	
6	0	0	0	
7	1 (0.9)	1 (1.2)	0	
Etiology of Heart Failure				
ischemic, *n* (%)	68 (64.2)	58 (67.4)	10 (50)	0.25
dilated, *n* (%)	36 (34)	27 (31.4)	9 (45)	
other, *n* (%)	2 (1.9)	1 (1.2)	1 (5)	
Implantation Strategy				
BTT, *n* (%)	4 (3.8)	4 (4.7)	0	0.59
BTC, *n* (%)	79 (74.5)	63 (73.3)	16 (80)	
DT, *n* (%)	23 (21.7)	19 (22.1)	4 (20)	
BMI, kg/m^2^	26.4 (IQR 7.6)	26.6 (IQR 7.7)	28.0 ±6.2	0.46
Body surface area, m^2^	2.0 ± 0.2	2.0 ± 0.2	2.0 ± 0.2	0.80
Comorbidities, *n* (%)				
Atrial fibrillation	31 (29.2)	26 (30.2)	5 (25)	0.64
Diabetes Mellitus				
oral therapy	26 (24.5)	22 (25.6)	4 (20)	0.52
insulin dependent	13 (12.3)	9 (10.5)	4 (20)	
History of Stroke	9 (8.5)	8 (9.3)	1 (5)	0.51
Arterial HT	38 (35.8)	32 (37.2)	6 (30)	0.55
Pulmonary HT	37 (34.9)	33 (38.4)	4 (20)	0.12
COPD	19 (17.9)	14 (16.3)	5 (25)	0.38
Dyslipidemia	35 (33)	30 (34.9)	5 (25)	0.40
Chronic renal failure	41 (38.7)	33 (38.4)	8 (40)	0.90
PVD	5 (4.7)	4 (4.7)	1 (5)	0.95
Cerebrov. disease	1 (0.9)	0	1 (5)	0.07
Redo case	28 (26.4)	23 (26.7)	5 (25)	0.87
Access, *n* (%)				
Full sternotomy	62 (58.5)	53 (61.6)	9 (45)	0.17
Less invasive strategy	44 (41.5)	33 (38.4)	11 (55)	
Creatinine, mg/dL	1.49 (IQR 0.8)	1.53 ± 0.5	1.72 ± 0.9	0.46
Total bilirubin, mg/dL	0.86 (IQR 0.8)	0.86 (IQR 0.8)	0.67 (IQR 1.4)	0.23
Albumin, g/L	35.2 (IQR 9.7)	35.6 (IQR 9.65)	33.9 (IQR 13.2)	0.26
PMAi L3, mm^2^/m^2^	942.6 ± 234.5	991.82 ± 207.1	618.15 (IQR 73.9)	<0.001

**Table 2:** Multivariate Cox proportional Hazards Model of 30-day Mortality. Abbreviations: PMAi, indexed Psoas Muscle Mass; FS, Full Sternotomy; LIS, Less Invasive Strategy.

Variables	Hazard Ratio	Confidence Interval (95%)	*p*-Value
Group PMAi			
high/moderate	Ref.	2.736–272.797	0.005
low	27.3		
Redo	29.8	1.894–467.644	0.016
Access			
FS	Ref.	0.000–9.073 × 10^199^	0.956
LIS	0.000		
Bilirubin	1.426	1.093–1.860	0.009

## Data Availability

The data presented in this study are available on request from the corresponding author.

## Appendix A

**Table A1:** Post-operative Adverse Events. *p*-Value calculated between high/moderate muscle mass and low muscle mass using *t*-test, chi-square test of Fisher’s exact test with Monte Carlo approximation, as appropriate. Data presented as *n* (%). Abbreviations: tach. Afib, tachycardic atrial fibrillation; ICU, intensive care unit; ECMO, extracorporeal membrane oxygenation.

Variable	Total Population (*n* = 106)	High/Moderate PMAi (*n* = 86)	Low PMAi (*n* = 20)	*p*-Value
Renal Dysfunction	20 (18.9)	15 (17.4)	5 (25)	0.526
Arrhythmias	33 (31.1)	28 (32.6)	5 (25)	0.928
Hemolysis	12 (11.3)	9 (10.5)	3 (15)	0.566
Hepatic Dysfunction	9 (8.5)	7 (8.1)	2 (10)	0.677
Hypertension	6 (5.7)	4 (4.7)	2 (10)	0.316
Major Bleeding				
requiring surgery	16 (15.1)	11 (12.8)	5 (25)	0.295
gastrointestinal	6 (5.7)	4 (4.7)	2 (10)	
both of the above	7 (6.6)	6 (7)	1 (5)	
Major Infection				
sepsis	17 (16)	14 (16.3)	3 (15)	1.0
driveline	1 (0.9)	1 (1.2)	0	
local	2 (1.9)	2 (2.3)	0	
Neurological Dysfunction				
ischemic stroke	7 (6.6)	6 (7)	1 (5)	0.9
hemorrhagic stroke	0	0	0	
other	17 (16)	13 (15.1)	4 (20)	
Pericardial Fluid Collection	12 (11.3)	9 (10.5)	3 (15)	0.694
Psychiatric	26 (24.5)	20 (23.3)	6 (30)	0.568
Respiratory Insufficiency	24 (22.6)	19 (22.1)	5 (25)	0.772
Right Heart Failure				
right heart decompensation	4 (3.8)	3 (3.5)	1 (5)	0.729
ECMO implant	2 (1.9)	2 (2.3)	0	
Venous Thromboembolism	2 (1.9)	1 (1.2)	1 (5)	0.343
Wound Dehiscence	4 (3.8)	2 (2.3)	2 (10)	0.161
Readmission ICU	12 (11.3)	10 (11.6)	2 (10)	1.0
Other Complication	19 (17.9)	15 (17.4)	4 (20)	0.754
